# The experiences and challenges of community health volunteers as agents for behaviour change programming in Africa: a scoping review

**DOI:** 10.1080/16549716.2022.2138117

**Published:** 2022-10-31

**Authors:** Mary Ndu, Ellena Andoniou, Sorcha McNally, Francisco Olea Popelka, Marisa Tippett, Elysée Nouvet

**Affiliations:** a Faculty of Health Sciences, University of Western Ontario, London, ON, Canada; b Faculty of Health Science, Western University, London, ON, Canada; c Department of Pathology and Laboratory Medicine, Department of Epidemiology and Biostatistics, Schulich School of Medicine & Dentistry, Western University, London, ON, Canada; d Research & Scholarly Communications Librarian, Western Libraries, Western University, London, ON, Canada

**Keywords:** Sustainable development goals, lived experiences, low-middle-income countries, human resources for health, universal health coverage

## Abstract

Community health volunteers are considered a vital part of the community health structure in Africa. Despite this vital role in African health systems, very little is known about the community health volunteers’ day-to-day lived experiences providing services in communities and supporting other health workers. This scoping review aims to advance understanding of the day-to-day experiences of community health volunteers in Africa. In doing so, this review draws attention to these under-considered actors in African health systems and identifies critical factors and conditions that represent challenges to community health volunteers’ work in this context. Ultimately, our goal is to provide a synthesis of key challenges and considerations that can inform efforts to reduce attrition and improve the sustainability of community health volunteers in Africa. This scoping review was conducted using the Preferred Reporting Items for Systematic reviews and Meta-Analyses extension for scoping reviews checklist to achieve the objectives. A comprehensive search of six databases returned 2140 sources. After screening, 31 peer-reviewed studies were selected for final review. Analytical themes were generated based on the reviewers’ extraction of article data into descriptive themes using an inductive approach. In reviewing community health volunteers’ accounts of providing health services, five key challenges become apparent. These are: (1) challenges balancing work responsibilities with family obligations; (2) resource limitations; (3) exposure to stigma and harassment; (4) gendered benefits and risks; and (5) health-system level challenges. This scoping review highlights the extent of challenges community health volunteers must navigate to provide services in communities. Sustained commitment at the national and international level to understand the lived experiences of community health volunteers and mitigate common stressors these health actors face could improve their performance and inform future programs.

## Introduction

The global shortage of human resources for health (HRH), especially in low-middle-income countries with limited health resources, represents a fundamental challenge to health equity. This shortage directly limits the achievement of Sustainable Development Goal (SDG) 3, which aims to ensure healthy lives and promote well-being for all, at all ages, and hinders progress on Target 3.8: achieving universal health coverage [1–4]. Strategies that countries continue to adopt and implement to address the shortage of HRH include task shifting and the introduction of various cadres of community health workers, including community health volunteers (CHVs) [5–7]

The World Health Organization (WHO) formally describes CHVs as lay health workers who are not professionally trained as healthcare professionals but have been trained to promote health within the community in which they reside [8,9]. To this definition, one can add that CHVs are community members that may or may not be elected by the populations they serve, who willingly collaborate and partner with governments, non-governmental organizations (NGOs), and others to serve members of their community without any form of compensation [10] CHVs may receive support from their government or NGOs working within the community. However, where provided, this rarely extends beyond stipends for transportation and miscellaneous expenses as an incentive for volunteering [11–13] While different health systems identify these unpaid health volunteers by different nomenclature (e.g. community health workers, community health extension workers, community health influencers and promoters, village health workers), for this scoping review, we use CHVs in reference to all community health volunteers who do not receive any wages or salary from the government, and are distinct, if not complementary, to formal community health workers who are government employees within a country’s health system.

CHVs have become vital to achieving universal health coverage on the African continent [1,4,9,14]. Currently, and while varying from context to context, CHVs may be responsible for basic community drug distribution, providing health information, diagnostics, prevention and treatment information, tracking and encouraging treatment and vaccination compliance, and recording and reporting births and incidences of morbidity and mortality [5,15,16]. As the first contact for many community members with national healthcare systems, programmes, and services, CHVs play vital roles in gaining community trust in, understanding of, and utilising available programmes and services aiming to improve health outcomes across the globe [17]. CHVs are widely recognised as enabling the extension of national and sub-national capacities for diagnosis, treatment, monitoring, and health promotion programmes [1,4,9,14].

Despite their crucial role in supporting African community well-being and health systems, little is known about the day-to-day work and challenges of African CHVs. Advancing an understanding of CHV experiences and challenges in their roles can provide governments and programmes relying on CHVs with important insights on how best to support CHVs in general and concerning specific performance expectations within community-based healthcare delivery or health behaviour change and promotion programmes. Hence, this scoping review aims to better understand the day-to-day experiences of a CHV in Africa. More specifically, this scoping review aims to identify critical factors or conditions that represent challenges to CHV work in Africa, analysing first-hand accounts of CHVs.

The following guiding questions informed this review. What are CHVs’ experiences of this work? Do CHVs feel (de)valued, and if so, by whom, under what conditions, and on what bases? Do CHVs feel they have the supports or capacity to do the work they are tasked to do? What do CHVs define as factors or environments (Administrative? Social? Technology-related? Training-related? Other?) that enable or hinder their ability to fulfill their CHV responsibilities? Do CHVs identify unmet needs or supports for fulfilling their responsibilities? How long do CHVs stay in these roles, typically, and what factors contribute to their departure from these positions when and if they do? Are the above experiences of CHVs gendered or otherwise distinct depending on the social identity or context of the CHV’s practice?

## Methods

The protocol for this scoping review was registered on Open Science Framework (OSF). The registration number is 10.17605/OSF.IO/2JZ7E, and the protocol preprint is also available on OSF Preprint at https://osf.io/hmy5t/. The team followed Tricco et al. Preferred Reporting Items for Systematic reviews and Meta-Analyses extension for scoping reviews (PRISMA-ScR) checklist statement [18].

### Literature search strategy

An academic research librarian, in consultation with the researchers, prepared a comprehensive search strategy and then conducted the literature searches in the following six databases: MEDLINE (Ovid), EMBASE (Ovid), PsycINFO (Ovid), CINAHL (EBSCOHost), Scopus, and Sociological Abstracts (Proquest). The searches were initially conducted between July 20 and 21, 2021, followed by a re-run of the searches on 5 November 2021, to ensure the most current articles were included in this review. Appropriate subject headings and numerous synonyms for the following main concepts were strategically combined and searched in this scoping review: Concept 1: All of Africa, Concept 2: Community health worker, Concept 3: Experiences/challenges. No filters or limits were used. Results were de-duplicated using Covidence, a systematic review platform. The complete search strategy can be found in Appendix A.

### Inclusion/exclusion criteria

The team developed and agreed on the inclusion criteria in dialogue with the research librarian on the team before commencing this review. Eligibility criteria included that studies be peer reviewed, published in English, and that these be focused on the experiences, challenges, motivations, and/or opinions of CHVs in Africa about their work, their role, and responsibilities in local or national healthcare systems, or in relation to specific programmes. No publication date limits were applied when searching the databases. Articles dedicated to description of salaried (as opposed to truly volunteer) CHVs were excluded. Conference abstracts, commentaries, non-English sources, and articles that did not include CHVs accounts of their experience were excluded.

### Selection of sources

The articles selection process involved three phases of screening using Covidence. The first stage involved title/abstract screening. The first and senior authors of this scoping review screened the same fifty articles independently in this first phase and compared sources they viewed as eligible for full review. Discrepancies on three out of 50 articles were discussed, and consensus reached on their inclusion/exclusion. During the title/abstract screening phase, articles which were not clear were retained for full-text review. The first author completed the full text review, consulting with the senior author when unsure about inclusion/exclusion. References of selected articles were also manually searched for relevance.

### Data charting

In extracting and analysing data from the sources, the team used Thomas and Harden’s proposed three stages of thematic synthesis: coding the data line-by-line according to its meaning, developing descriptive themes from the data, and generating analytical themes based on the reviewers’ interpretative construct of the descriptive themes. As an inductive approach, thematic synthesis provides a critical lens to generate high-level themes that capture the key messages from multiple qualitative data [19,20].

First, a data extraction template was developed to collate relevant data from the studies (see Appendix B for table of characteristics). This was done using a partial double extraction process to reduce errors during the process [20,21]. Two members of the team with moderate and substantial literature review experience independently developed data extraction templates based on a review of the same ten articles. This involved coding the data line-by-line according to its meaning and developing descriptive themes that could capture patterns each reviewer perceived within the data and that corresponded to the review objective of understanding the lived experiences and challenges of a CHV. The moderate and substantial compared extraction tables and reached a consensus on critical themes and definitions to guide subsequent data extraction. The minimally and moderately experienced reviewers respectively, divided up the remaining articles and completed the data extraction using the agreed-upon criteria and extraction table. The first author reviewed minimally experienced reviewer’s extraction for completeness and coherence, having read the articles. Additionally, the experienced reviewer reviewed the completed data extraction tables to ensure consistency in categorising extracts.

The final step involved meeting to discuss and agree on key analytical themes and messages for analysis. Analytical themes were generated beyond the descriptive themes outlined in the studies. To do this, each reviewer independently considered the themes and messages contained in the studies and generated themes based on their understanding. The results are presented in a thematic format with direct quotes from extracted interviews with CHVs in the studies reviewed.

### Data item

The team extracted data on the studies’ characteristics including country of origin, study type, year of publication, intervention area, study participants, contextual factors i.e. gender, participant role, education, years of experiences and study location.

## Results

### Selection of sources of evidence

**Figure 1.**
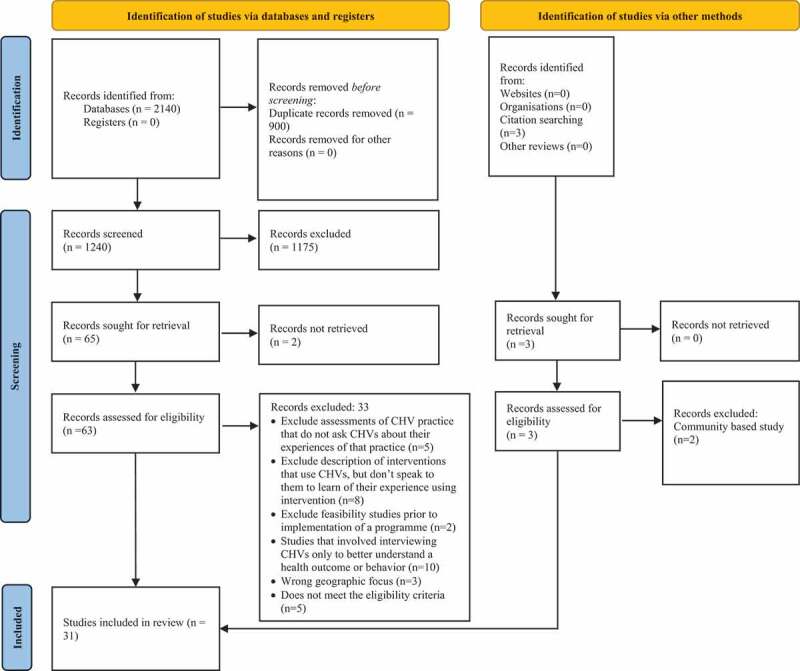
PRISMA diagram.

A total of 2140 articles were retrieved from database and other methods of searches. After removing duplicates, we identified 1240 articles for screening. During the title and abstract screening, an additional 1175 articles were excluded, with 65 retained for full text screening and retrieval. Of these, two articles could not be retrieved. Of the 63 assessed for full text screening, 33 were excluded for the following reasons: Exclude assessments of CHV practice that do not ask CHVs about their experiences of that practice (n = 5); Exclude description of interventions that use CHVs, but do not speak to them to learn of their experience using intervention (n = 8); Exclude feasibility studies prior to implementation of a programme (n = 2); Studies that involved interviewing CHVs only to better understand a health outcome or behaviour (n = 10); Wrong geographic focus (n = 3); Does not meet the eligibility criteria (n = 5). A total of 31 studies were selected for final analysis. Details of the sources are presented in the below PRISMA diagram Figure 1.

### Characteristics of sources of evidence

Sources retained contained a mix of studies (20 qualitative; 3 quantitative; 8 mixed quantitative/qualitative). Ten studies (32%) were health systems strengthening (HSS) interventions; 8 studies (26%) HIV/AIDS and TB; 5 studies (16%) maternal, newborn, and child health (MNCH); 2 studies (6.5%) palliative care and 2 studies (6.5%) malaria; while the remaining percentage was split between 1 study on family planning, 1 study on social and behaviour change communication (SBCC) and 1 study on eHealth at 3.1% each. One study did not mention an intervention. The studies were conducted in urban, semi-urban, and rural areas in African countries: Ethiopia, Kenya, Malawi, Nigeria, Rwanda, Sierra Leone, Liberia, Democratic Republic of Congo, South Africa, Tanzania, Uganda, Zambia, Mozambique. Participants in the studies reviewed included a mix of male and female volunteers. Most of the CHVs reported being married with children with volunteering experience ranging between five months to 20 years. Some of the studies reported on the educational level of the CHVs, with most of the CHVs having either completed primary or secondary level education. Two studies included some CHV participants with post-secondary education [22,23]. For more details on the study characteristics see Appendix B.

In terms of the focus of the studies, eight were conducted to examine volunteer health worker participation and performance in community interventions [22–28] while 17 examined the motivational factors that could improve the retention and performance of volunteers at the community level [29–50].

### Synthesis of results

Presented below are key themes that emerged from the studies that addressed the first-hand accounts of CHVs experiences and challenges in Africa.

#### Challenge of balancing responsibilities with family obligations

One common challenge that emerged across several studies, especially for female CHVs, was the challenge of balancing or managing CHV responsibilities alongside family obligations. Six of the thirty-one studies reported that family and spousal support play a crucial role in CHVs’ work-life [24,29–31,37,40,41]. Family obligations were noted as coming under pressure due to being a CHV, including income-generating activities to support the family, household chores, and spending quality time with children. Many CHVs underlined the particularly demanding nature of their volunteer role. They report that often, fulfilling CHV responsibilities could and did require responding to community members at all hours, including in the middle of the night and during meal preparation times. Findings show that volunteering can and often does result in neglecting income-generating activities. Thus, in two studies, CHVs who already have casual jobs, businesses, and farming as sources of income felt forced to make a choice between volunteering and earning income to support their families:

Even the time I spend going around households can be converted into loss income because I can use that time farming or selling produce … .but I cannot think like that because I’m passionate about improving my community [41, p.5]

My potato frying business [is] in the evening or making porridge in the morning. … If I am needed somewhere, my business has to close. … those potatoes usually get spoilt; they won’t find another person to sell them [FGD 6–KALOLENI] [31, p. 6]

My child needs school fees but I say wait for a while. If you see patient, I use my money. I don’t know where God is. We don’t have job. Now, we have a problem because cost of life is high and there is no job.’ [Female, CHW3, Group 2] [33, p. 842]

Some CHVs reported feeling discontent and disapproval from families for spending so much time on volunteer work when they could engage in more profitable jobs [24,29]. Findings indicate that CHVs, especially women, are expected to maintain their roles in the family while volunteering. The following quote, taken from a study with CHVs called Mentor Mothers in the study’s country context, provides a window into the sorts of tension on the home front CHVs may need to navigate alongside their community health work:

Another Mentor Mother [MM] shared tensions that had arisen in her household, where she as a young wife (makoti) was expected to plan meals and clean. After accompanying a client to an emergency clinic visit, she recalled, “I arrived at home past eight, and when I got home, I saw the mood had changed, but I told myself that what matters is I helped the baby” (MM6) [25, p. 1255]

Several CHVs reported struggling with creating a work-life balance, and feeling they are neglecting their families’ needs to fulfill their CHV responsibilities. Findings reveal female CHVs are particularly torn between their commitments to their CHV role, and their socially normative role as homemakers. Female CHVs’ narratives indicate that many face a triple burden of care because of their CHV positions, integrating CHV work alongside income-earning activities, while still being the person in their household with primary responsibilities for domestic work. A quote from one CHV in Kenya saying:

For now, I have a grandchild whom I am taking care of. My challenge is, I have to look for someone [to] leave my grandchild with before I can go to the community. Sometimes I leave her [childcare] and tell her, ‘I will give you anything that I get [payment]. Or sometimes when I am called for a seminar and I leave the same person [childcare] with my grandchild, she also hopes to get something [to be paid]. [FGD 4–RABAI.] [31, p. 5]

Some CHVs reported family members playing a role in supporting individuals’ fulfillment of CHV responsibilities by providing moral support, and by taking on an extra farm or domestic work, including responsibilities for cooking, washing clothes and rearing children in the case of women. CHVs reported how the need to improve their family’s economic status and support the family financially often competed with their volunteering. The reported unremunerated nature of the job made it hard for these CHVs to provide necessities for their children, as exemplified by the following quote from a study in Kenya:

At the end of the month, the [CHVs] were not paid enough, and they had been working. They, therefore, could not supply their families with some of their basic needs, so they decided to leave the job. (2013, Female FGD 2, Eldoret West). My children can’t get the basic needs like soap and you can’t tell the community about being clean when the CHV is not clean. (2015, Female, FGD 3, Eldoret West) [30, p. 97]

Where CHVs reported an inability to provide what they regarded as sufficient financial support to their family due to their CHV time commitments, this was reported as stressful. The experience of primary responsibilities being frequently neglected because of CHV duties also emerges in the literature as resulting, at least for some CHVs, in feelings of hopelessness and mental stress:

Now at least three times in a week, I wake up [in the middle of the night]. I think about my family, about supporting them with a good job. I will not sleep until the next day. There is no happiness with me. When I can’t sleep, I will feel depressed all day. [38, p. 56]

One MM described ‘leaving the pots on’ after she had started making dinner to put on her uniform and go help a client who had called with an emergency. You don’t ever think that you supposed to tell the manager that today, I left work at five, the only thing you think is that I have to help this person, [it] doesn’t matter what time I knock off, I’m not supposed to say I’m rushing for 14:00, rather this person die because you want to knock off at 14:00 (MM6). [25, p.1253]

Some CHVs noted that pressures on income-earning activities from CHV work were offset somewhat by the provision of support in the form of stipends for transportation and materials [29,30,41]. Other CHVs especially men reported and appreciated receiving payments and in-kind support from community members, for example in the form of food and help with farm work, in exchange for services they provided [31].

#### Limited resources

Financial and logistical challenges such as non-remuneration, stipends for transportation, stock out of medicines, lack of uniform and badges facilitating their identification as health workers, and lack of training to provide additional services needed in their communities, were among the critical challenges CHVs reported encountering in their roles [23,24,26–31,34,36–38,40,42,45]. Many volunteers especially reported motivation to volunteer and improve their knowledge as linked to a commitment to helping their families and community. Although, male CHVs reported aspiration to move to higher positions in government offices, NGOs, or political offices at the local level as the motivation to work as CHVs. However, and related to CHV responsibilities and family conflicts noted above, many CHVs also reported the absence of stipends or payments that might offset the time they commit to CHV work as constituting a significant challenge. In one study in Zambia CHVs reported:

Our stipend is too little, and we sometimes spend three months without receiving it.’ [(47-year-old Setswana-speaking female from JS Moroka sub-district)] [43, p. 417]

In another study in Ethiopia, CHVs reported:

We have to take our children to school, but we are not after money more than we want to help the community. But we need money for crèche [daycare] [49, p.146]

Studies reported that the stress produced by a lack of resources available to CHVs to fulfill their responsibilities extended beyond the issue of stipends [41,48], and that some CHVs have incurred additional financial burdens resulting from wanting to provide care to community members without resources being allocated to them to do so. CHVs reported, for example, providing food items to patients and spending personal funds to buy materials such as lamps, kerosene, candles, and torches/flashlights. In South Africa, one CHV notes:

You have to share that little amount you are earning, to give a client to buy some bread and at some point, we used to give some soups to the patients from our own families to help them, just because we have a conscience, they do not have food and we cannot report it to our seniors as they will tell you that there is no food to give to the clients … . Sometimes we do not have …. [Olga, Female, 30] [42, p. 5]

Studies also showed that stock out of medicines and inconsistent supply of resources affected the CHVs’ performance and effectiveness in providing services to their communities. In some communities, CHVs were placed in unpleasant and frustrating positions because they were perceived as withholding medicines and materials from needy communities. So much so, that there were times when the community would not trust the CHV:

People always ask me why we don’t receive medicine, yet we got this box. It aﬀects me because we are the immediate health workers who have to give ﬁrst aid to the people, so it frustrates me and them when there is nothing to give! (Male CHW, 67 years old) [39, p. 393]

In some settings, CHVs reported having limited access to opportunities to develop their skills. According to studies, CHVs might be expected to limit their efforts to basic health promotion and disseminating non-prescription medications, such as family planning methods. However, they often had to assume additional health service responsibilities in practice since they are often the only healthcare providers in remote areas. CHVs reported wanting further training, for example, to provide more comprehensive information and services inclusive of non-communicable diseases like cancer and diabetes and detecting gender-based violence. CHVs in one study in Uganda state:

We appreciate the trainings we have had on child health, but we don’t get enough in other diseases like diabetes, eye disease, cancer … and there is a weakness in supervision. [Male CHW, 45 years old] [39, p. 391]

After initial CHV training, it appears that there is a lack of refresher courses. This lack suggests there is no appreciation, or little appreciation towards the CHVs contributions and in turn, discourages some CHVs from continuing with the programme [51]. As Kweku et al. [51] note:

Such CHMC members were frustrated that their skills have not been upgraded over the years and thus were not seen as important stakeholders in the CHPS initiative [p. 9]

In another study in Kenya, digital literacy was a challenge for CHVs in a mHealth intervention. However, the CHVs reported additional structural challenges like weak internet coverage, poor power supply, and slow replacement of tools:

… it has a negative because at the moment, okay there is a time I lost my phone. It was stolen and it had that line of (organisation name) and it took a lot of time 3 months for them to return for me the line, so there is no data I have been checking. Because you cannot check without bundles, that’s one thing because if I check for that one that desktop in the office, that one the bundles you find that it’s not even there. Another thing since they returned for me that line, it was last month I have not been able to access any data because the password I am using and anything it doesn’t open it keeps telling me your password is wrong or your password is wrong every time every time. [KII CHA] [23, p. 7]

#### Stigma and harassment

CHVs reported personal experience with stigma and harassment from both facility health workers and community members [28,33,45]. This was also a challenge of the work, in some settings. Where such stigma and harassment emerged, the interprofessional collaboration between CHVs and other health workers indicated a fragmented relationship, with CHVs feeling they were receiving little to no respect for their work. Some CHVs in Nigeria note:

On ANC days, we work together, do everything together. But if there is anything [of benefit], they will say, leave, are you one of the staff? … when it is time to share they will say it’s for the staff … when they see a positive mother it is then that they remember us.—[Intervention MM FGD3] [29, p. 5]

CHVs described feeling stigmatised by fellow health care workers who treated them differently because they were not formal health workers within the health facilities. Some CHVs identified training, educational qualifications, and recruitment status as factors influencing other health workers’ rejections and discrimination:

We don’t have ID cards, no uniforms…. [HCWs] said we don’t work with a certificate and are not members of staff. Even if we are staff, we are not learned.—[Intervention MM FGD3] [29, p. 8]

Some of the CHVs described similar experiences of being ignored and disrespected by other health other workers within health facilities. One study in Nigeria reported;

In some instances, even the lowest-cadre clinic staff wielded illegitimate authority over MMs, further distracting them from their primary duties: … The attendants are ordering us about, telling us we have not done this or that.— [Control Group FGD 3] [29, p. 8]

Some CHVs reported toxic relationships between volunteers and supervisors. Some CHVs reported supervisors and health workers yelling and using abusive words if they did not perform tasks in the facility. This greatly affected the mental health of the CHV:

The last matron we had in Facility A, she gave me a ‘heart attack’. As soon as I get to the gate of the clinic my heart always skips because I know it will be trouble all through … But with this new one, I do not have any problems.—[Intervention MM FGD3] [29, p. 5]

Some CHVs reported supervisors are devaluing and excluding them from activities that could provide some funds. A CHV in Kenya explains:

You will find that [the supervisor] will only invite the few secretly [to receive some compensation] and not all the CHWs. (…) This is something that makes us demotivated and even think of withdrawing because he is not transparent. (Voluntary CHW, FGD-Kenya) [46, p. 7]

Some HIV-positive CHVs reported being discriminated against by the community and health facility health workers. Similarly, female CHVs in one study were subjected to name-calling and insults from community members when the job involved working on reproductive health interventions:

The former in-charge didn’t even allow us to come close to his office; he sent us away as soon as we got close because we are HIV-positive. But the new one we have now doesn’t discriminate.—[Intervention MM FGD1]. Some of the nurses at my site stigmatize in the way they treat us. They treat us as if it is because of being wayward that we have HIV.—[Intervention MM FGD1] [29, p. 8]

In another study, female CHVs reported being harassed by community members because of the intervention focus area they work. The author explains: *‘the CBMs reported that some community members ridiculed them by claiming they were prostitutes, which seemed related to their task of promoting contraception, including condoms.’* [34, p. 4]

#### Potential gendered benefits and gendered risks

Studies reported that creating community health worker volunteers can address existing gender inequality by empowering women through health [29,36,46,48]. As Closser et al. [36] note for the context of Ethiopia, the CHV programme was *‘ … designed to encourage women to leave the house and gain decision-making power vis-a-vis their husbands – and to use this power to achieve specific, state-mandated, domestically centred goals.’ (p. 298)*

However, at least one study suggests that recruitment preferences may favour unmarried or divorced women because of the high turnover of a younger demography who craved more financial freedom [29]. Studies reported gendered differences in the motivation to volunteer, often closely associated with the desire to improve their families’ financial situation [23–25,28,29,31,33,35,37,39,44–48,52]. Although women also expressed the desire for career advancement in connection to work experience acquired as CHVs [27,31,36,46,48], the kinds of future careers they hoped to get with minimal education is not reported in the literature. More male CHVs interviewed were explicit and detailed in connecting their motivation to volunteer as being linked to providing financially for their families and to specific career advancement aspirations, such as finding paid employment with the Ministry of Health or NGOs, with some harbouring political ambitions at the grassroots level [26,35,38,45,46].

Male and female CHVs in at least six studies described their frustration and disappointment with the inability to achieve their desired financial objectives [23,25,31,33,39,45,46,48]. Some female CHVs reported frustration and disappointment in the CHV scheme. They did not feel they could achieve their career or financial objectives through the volunteer programme. As one study notes in Ethiopia, *‘Empowerment, defined as significant changes in social power and economic status, did not accrue to WDA leaders [Women’s Development Army] through the programme. Neither … was able to advance themselves economically through WDA work.’* [37, p. 304]

In contrast, at least some male CHVs regarded volunteering as a pathway to higher positions, with many reporting being able to access paid employment opportunities because of their volunteer experience. In a study in Tanzania, one CHV states:

What drew me to this work was my love for the Ministry of Health. I thought I would be lucky to join the ministry, and by good luck, I am in the ministry [through my CHW position]. I am grateful for this opportunity (CHW1, age 35, male) [32, p. 7]

Some female CHVs described feeling unsafe while volunteering [23,24,31,35,43]. Without provisions for transportation, they describe safety concerns with risky commutes to work and assigned households. Female CHVs also described being attacked and fear walking alone on the streets while providing services in the context of South Africa:

… they walked long distances to households (76.3%) and did not feel safe to walk on the streets or to interview patients with mental health problems [43]

#### Health system challenges

CHVs considered the absence or lack of standardised remuneration for their work as a significant challenge but also as a failure of the health systems they served. Many CHVs reported, sometimes with clear disappointment or frustration, government promises to provide payment for volunteering and not fulfilling it [26,28–30,33,34,36–40,45,46,50,53]. Where CHVs reported not receiving money or less than what the government promised as an incentive, this can lead to feelings of distrust towards the government:

This subsidy is just not enough for anything, but they promised us and should at least give us the little at the end of the month, and they give just nothing. (…) I have to support my family.’ (Voluntary APE – Agente polivalente elementar (elementary multipurpose agent), SSI-Mozambique). ‘This job is very hard to do (…). This payment we are supposed to get, the government is the one that is not trustworthy. They should give some money for us to be paid every month.’ (Voluntary CHW, FGD-Kenya) [46, p. 7]

Some studies reported partial financial incentives that stopped once the NGOs were no longer within the community [29,31,33,36–38,40,46,50]. Non-government funding of programmes delivered by CHVs does facilitate Ministries negating responsibility for remunerating CHV work in some settings:

Like this centre gets 1,200 kwacha for general maintenance at the health post every month. We just asked them to give us part of the money since we have delays in our salaries. We needed the money to repair our bikes. But they said no, this is programme is not under the Ministry of Health, you are sponsored by Clinton Health Access Initiative (CHAI), so go and ask CHAI for the money.’ (CHA 6) [47, p. 8]

Some CHVs reported increased and unclear scope of work, and performing clinical tasks within their supervising primary health centre (PHCs) [28,40,50]. CHVs reported unclear division of labour as they are often required to perform certain tasks usually ascribed to health facility workers (HCWs), such as administering drugs and filling out facility registers which are outside their scope of work. These unclear descriptions of roles and responsibilities made it difficult for CHVs to collaborate with their colleagues in the facilities effectively:

I don’t know exactly what registers we are supposed to handle and those we should not be responsible for, because we get conflicting information … This clarification will make me focus on those registers that are my responsibility and reject any unrelated tasks.—Intervention MM FGD1. Sometimes, they [HCWs] make us do jobs that are not part of our responsibilities as peer counselors… They give us additional jobs apart from the peer counseling job.—Control MM FGD3 [29, p.6]

Moreover, CHVs in several contexts noted the inequity of their non-remuneration in light of their counterparts and supposed colleagues in health facilities being provided with a monthly maintenance allowance [28,29,36–38,40,45,50].

## Discussion

Building up population health relies on governments, communities, private sectors and other stakeholders understanding how one of their most valuable and important human resources for health (HRH), CHVs, experience their lived realities day-to-day.

This review adds to existing knowledge of CHVs, using personal accounts of their daily experiences to highlight the challenges they face while providing services within their communities. The findings outline tensions and challenges that CHVs face in their daily roles. Two critical findings were revealed in this review. First, there are potential gendered differences in the experiences of male and female CHVs. From the studies reviewed more women than men are often recruited as CHVs which could be an indication of a recruitment bias. Some studies have arrived at the same conclusion that women are often the preferred gender for the role of CHVs [54–56]. Secondly, the commitment of CHVs to continue building their communities’ health despite their need for work-life balance, resources, monetary compensation, training, and better supervision.

The consistency with which CHVs report struggling with balancing their work and personal life responsibilities is reason for concern and merits addressing. There is an indication that communities and institutions that promote CHV’s value and rely on their labour appreciate their work. However, there was no indication that their physical, psychological, and economic well-being were adequately considered during implementation. Similar studies have found that CHVs, while providing service to community members, also provide mental support, often to the detriment of their well-being [54–57].

Additionally, CHVs may face critique at home from family members who may not understand or are unwilling to support this work without material or financial gain [58], alongside these worries, and alongside facing critique from interprofessional collaborators (i.e. supervisors, healthcare professionals), and in some instances suffering castigation. This review’s findings align with similar studies on community health volunteers. For example, a similar multi-country review found that community health volunteers, especially women, were often criticised for their role as volunteers by their spouses, family, and co-workers [59]. From our findings, male CHVs often had different motivations and aspirations for becoming CHVs, with many moving on to high positions within the community. Male CHVs, we also found, experienced limited household burdens or mental health distress due to the needs of the community members they serve. These findings are not unique to the sub-Saharan context. A comparative study of CHWs in Brazil reached similar findings – that women were more likely than men to experience mental health distress and household burden due to work-related reasons [60]. If national and global commitments to expanding healthcare services at the community level continue to grow, in line with commitments to the Sustainability Development Goals, these commitments will likely only further increase pressures on CHVs, including potentially gendered pressures. Ensuring recognition for the workloads CHVs assume and developing supports to mitigate the diverse challenges CHVs may face in their work is crucial to sustaining the well-being of these key healthcare actors.

As we reflect on CHVs roles and interactions, it is vital to remember that CHVs are not a homogenous group. CHVs in sub-Saharan Africa as elsewhere are diverse in their status and gender identities, particularly over time, space, and place [14]. This does have implications for ensuring CHVs are adequately supported in their work in specific settings. For instance, evidence supports that approximately 70% of CHVs are women in sub-Saharan Africa [61]. With this current demographic reality, it is critically important to acknowledge the triple burden faced by women because of their triple role in society (reproductive work, productive work, and community management work), which is, of course, in addition to caring for their personal well-being and health (e.g. illness episodes with malaria, TB or HIV/AIDS). It is clear from CHV narratives that the majority of CHV work is being conducted in settings where women traditionally hold primary responsibilities for childcare, food preparation, and general maintenance of the household. This results in gendered implications for CHV experiences of balancing CHV roles with household responsibilities. Any initiatives introduced to support CHVs in their work will benefit from considering how such support may need to be different for men and women in these roles, in particular settings.

While challenges balancing family and gendered responsibilities were prominent in the literature reviewed, it is equally important to highlight the ways in which challenges of being a CHV in sub-Saharan Africa arise in relation to the healthcare system more generally. Our review makes evident that CHVs are rarely well integrated into primary care teams, and their potential impact is limited where there is an inability to follow up on patients and health needs due to limited resources. Many studies highlight the crucial role CHVs play in primary healthcare to address complicated barriers to care [1–12,23,62,63]. Their contributions are especially important towards fulfilling the United Nations Sustainable Development Goals (SDGs) [44,54,55]. It is clear from the experiences and accounts reviewed that CHVs in Africa are exposed to a myriad of psychosocial and physical stresses because of their stark encounters with mortality, morbidity, disability while often unable to comprehensively help those who are suffering due to the limited availability of resources (i.e. materials, services, medicines, training, etc.). Even as they describe such realities, CHVs come across in the literature as deeply devoted to their work and communities.

Some CHVs in the reviewed studies went so far as to share their income and resources – e.g. food and money – with community members as they perform their care duties to ‘do what is right.’ This deep sense of responsibility to help their communities and fulfill their duties of care is juxtaposed against concerns: for the well-being of those they serve, for the strain on their time to care for their own families and household responsibilities which put some of them at a constant crossroads. Optimising the impact and contribution of CHVs to the SDGs and sustaining the well-being and energy of CHVs in sub-Saharan Africa, requires finding ways to increase their reliable access to resources needed to carry out the health activities they are expected and motivated to undertake.

This review highlights the tensions that can emanate from a poor understanding of the CHVs role, responsibilities, and scope of work, between the CHVs and other cadres of health workers. There seems to be an undervaluing of their work at the system level among healthcare professionals. Within health systems, CHVs frequently lack a voice and agency to control their circumstances and carry out their work as they would like to do. This lack of control can potentially undermine beneficial relationships between the CHV, the health system and the communities they serve. It may also contribute to decreased motivation, job satisfaction and eventually ineffectiveness.

As more countries prepare to shift their acute focus from challenges with infectious diseases to non-communicable diseases and confront various forms of violence, mental health, and injuries, the CHV portfolio will inevitably expand to include health promotion, prevention, and services in these areas. Threatening to further overload CHVs and progressively blurring the lines of their roles, responsibilities, and scope of work in increasingly complex ways. Under the banner of global health, achieving health for all is accomplished at the expense of underpaid or non-remunerated volunteers.

Based on the findings of this scoping review, we recommend that governments take steps, if these are not already under way, to review CHV supports and programmes in their jurisdiction. Ideally, CHVs would have the opportunity to regularly share their experiences and suggestions. In dialogue with CHV representatives (including male and female CHVs), Ministries of Health may then identify ways to ensure the important work CHVs do, can be more effective and less draining to CHVs in specific national and sub-national contexts. This does mean investment in human resources for health planning and earmarking some funds to work with CHVs. Including identifying recommendations in collaboration with CHVs that can support the long-term effectiveness, success and sustainability of programmes, as well as CHV retention. Ministries of Health may explore regularising CHVs as a cadre and consider standardising their training and scope of responsibilities. Some governments may be ready to consider provision of salaries or stipends to CHVs. Additional considerations should also include ongoing supports essential to their supervision and provision of resources needed to deliver services, as well as basic mental health support for CHVs.

CHVs are a critical group of healthcare workers connecting communities and health systems. They are citizens and often members of communities with complex responsibilities beyond their CHV commitments, yet their invisibility in global and national conversations about how the SDGs will be achieved casts them as nothing more than delivery mechanisms of health programmes. Themes emanating from this review point to the need for future research to focus on gaining an improved understanding of their experiences as they relate to applying advanced behaviour change interventions to their practice as part of their responsibilities at the individual, household, and community levels. A step further in the research would allow practitioners, decision, and policy makers to concentrate on approaches that give agency, encouragement, and support to CHVs to participate in the design and implementation of these programmes. Allowing them to share their knowledge about activities and approaches that work in and with the communities they serve. Understanding CHVs’ insights, experiences, and programmatic implications on their daily realities is key to achieving the greatest impact and sustainability.

### Strengths and limitations

Strengths of this review include its attention to lived experiences of community health volunteers, and its incorporation of quotes from CHVs to illustrate themes. Inclusion of CHVs accounts of their day-to-day work and challenges supports a nuanced and detailed attention to CHV experiences that has not previously been available in reviews of CHV work in sub-Saharan Africa. This is the first synthesis of the experiences of CHVs posing challenges in their day-to-day life as health volunteers in Sub-Saharan Africa. It provides essential insight into the mental state of volunteers and the struggle to balance their needs with those of the community members they serve. The findings demonstrate how efforts to achieve the SDGs could be undermined by neglecting the CHVs, given their relationship and proximity to the community. It is also a review attention to gendered differences in experiences and challenges of CHVs: understanding these differences can inform effective supports to CHVs.

This review had some limitations. First, we included only studies in English due to the language fluency limitations of our team. The authors are confident that the findings reported in this review provide an accurate picture of CHVs’ experiences in Sub-Saharan Africa, but inclusion of Arabic and French sources might have enabled this review to include North Africa as well. Secondly, this review did not include grey literature. While publication bias is often avoided by accessing grey literature such as theses, dissertations, conference papers, and programme reports [64], the focus of this review was to analyse the personal accounts of CHVs and not the opinions and perspectives of other cadres of health workers or those who make decisions. Therefore, the authors decided to include only peer-reviewed studies with qualitative data reported directly from CHVs, although, grey literature informed the framework and discussions.

Lastly, the eligibility criteria of this scoping review are inclusive of publications capturing the lived experiences of CHVs across the gender identity spectrum. Nonetheless, gender roles, expectations and norms both socio-culturally and across multiple levels of the health system, more women than men work as CHVs, ultimately capturing the voices of more participants identifying as women.

## Conclusion

CHVs play a vital role in dynamic and complex communities. Understanding the experiences and challenges CHVs face can inform governments and programmes on how best to support community health volunteers both generally and specifically with respect to performance expectations within community-based healthcare delivery or health behaviour change and promotion programmes. This scoping review points to the need to understand the inequities CHVs face, the power imbalances they encounter in their labour relations and interactions with other healthcare providers, supervisors, care recipients and family members, which collectively shape their lived experiences. Of note are the gendered nature and risks faced by women taking on these positions to serve their communities. We need to recognise the unique challenges of female volunteers especially given the focus on gendered experiences of female health workers globally. Future research on CHVs may focus on understanding the potential mental health implications of the volunteering experiences on CHVs motivation, performance and the types of mental health supports that could be offered to CHVs. Finally, studies on the gender equity implications of focusing on women as CHVs need to be explored further. Ultimately, contributing to the sustainability of this under-considered cadre of healthcare workers is crucial to healthcare delivery in many countries.

## Paper context

Community health volunteer workers have significantly contributed to improving health in low- and middle-income countries. Past studies have focused on the importance and performance of community health volunteer workers. However, there is limited focus on their daily personal experiences and challenges. This review clarifies perceptions, challenges, and overall experiences of community health volunteer workers explicitly noted as influenced by social identities or context of practice in the literature. Findings indicate community health workers face various daily challenges, including work-life balance and financial constraints.

## Abbreviations

CHA: Community Health Assistant
CHV: Community Health Volunteers
CHW: Community Health Workers
HCW: Health Care worker
HIV/AIDS: Human Immunodeficiency Virus/Acquired Immunodeficiency Syndrome
HSS: Health systems strengthening
HRH: Human Resources for Health
MNCH: Maternal, newborn, and child health
MM: Mentor Mothers
NGOs: Non-governmental organizations
OSF: Open Science Framework
PHCs: Primary Health Care Centers
PRISMA ScF: Preferred Reporting Items for Systematic reviews and Meta-Analyses extension for scoping reviews
SBCC: social and behaviour change communication
SDG: Sustainable Development Goals
TB: Tuberculosis
WHO: World Health Organization

## Appendix A: Search strategy

Ovid MEDLINE Search History

(R) ALL <1946 to November 04, 2021>

1. sub-saharan africa.mp. or exp “Africa South of the Sahara”/
2. exp Africa, Western/or exp Africa, Central/or exp Africa, Eastern/or exp Africa, Southern/
3. “sub saharan africa”.mp. [mp=title, abstract, original title, name of substance word, subject heading word, floating sub-heading word, keyword heading word, organism supplementary concept word, protocol supplementary concept word, rare disease supplementary concept word, unique identifier, synonyms]
4. “africa south of the sahara”.mp. [mp=title, abstract, original title, name of substance word, subject heading word, floating sub-heading word, keyword heading word, organism supplementary concept word, protocol supplementary concept word, rare disease supplementary concept word, unique identifier, synonyms]
5. (Angola or Benin or Botswana or “Burkina Faso” or Burundi or Cameroon or “Cape Verde” or “Central African Republic” or Chad or Comoros or Congo or “Cote d’Ivoire” or Djibouti or “Equatorial Guinea” or Eritrea or Ethiopia or Gabon or Gambia or Ghana or Guinea or Guinea-Bissau or Kenya or Lesotho or Liberia or Madagascar or Malawi or Mali or Mauritania or Mauritius or Mozambique or Namibia or Niger or Nigeria or Reunion or Rwanda or “Sao Tome and Principe” or Senegal or Seychelles or “Sierra Leone” or Somalia or “South Africa” or Sudan or Swaziland or Tanzania or Togo or Uganda or “Western Sahara” or Zambia or Zimbabwe).mp. [mp=title, abstract, original title, name of substance word, subject heading word, floating sub-heading word, keyword heading word, organism supplementary concept word, protocol supplementary concept word, rare disease supplementary concept word, unique identifier, synonyms]
6. exp Angola/
7. exp Benin/
8. exp Botswana/
9. exp Burkina Faso/
10. exp Burundi/
11. exp Cameroon/
12. exp Cape Verde/
13. exp Central African Republic/
14. exp Chad/
15. exp Comoros/
16. exp Congo/
17. exp “Democratic Republic of the Congo”/
18. exp Cote d’Ivoire/
19. exp Djibouti/
20. exp Equatorial Guinea/
21. exp Eritrea/
22. exp Ethiopia/
23. exp Gabon/
24. exp Gambia/
25. exp Ghana/
26. exp Guinea/
27. exp Guinea-Bissau/
28. exp Kenya/
29. exp Lesotho/
30. exp Liberia/
31. exp Madagascar/
32. exp Malawi/
33. exp Mali/
34. exp Mauritania/
35. exp Mauritius/
36. exp Mozambique/
37. exp Namibia/
38. exp Niger/
39. exp Nigeria/
40. exp Reunion/
41. exp Rwanda/
42. “Sao Tome and Principe”/
43. exp Senegal/
44. exp Seychelles/
45. exp Sierra Leone/
46. exp Somalia/
47. exp South Africa/
48. exp Sudan/
49. exp Swaziland/
50. exp Tanzania/
51. exp Togo/
52. exp Uganda/
53. exp Zambia/
54. exp Zimbabwe/
55. Africa.mp. or exp Africa/or exp Africa, Northern/
56. (“Northern Africa” or “Southern Africa” or “Western Africa” or “Eastern Africa” or “Central Africa”).mp. [mp=title, abstract, original title, name of substance word, subject heading word, floating sub-heading word, keyword heading word, organism supplementary concept word, protocol supplementary concept word, rare disease supplementary concept word, unique identifier, synonyms]
57. (Algeria or Egypt or Libya or Morocco or Tunisia).mp. [mp=title, abstract, original title, name of substance word, subject heading word, floating sub-heading word, keyword heading word, organism supplementary concept word, protocol supplementary concept word, rare disease supplementary concept word, unique identifier, synonyms]
58. exp Algeria/
59. exp Egypt/
60. exp Libya/
61. exp Morocco/
62. exp Tunisia/
63. 1 or 2 or 3 or 4 or 5 or 6 or 7 or 8 or 9 or 10 or 11 or 12 or 13 or 14 or 15 or 16 or 17 or 18 or 19 or 20 or 21 or 22 or 23 or 24 or 25 or 26 or 27 or 28 or 29 or 30 or 31 or 32 or 33 or 34 or 35 or 36 or 37 or 38 or 39 or 40 or 41 or 42 or 43 or 44 or 45 or 46 or 47 or 48 or 49 or 50 or 51 or 52 or 53 or 54 or 55 or 56 or 57 or 58 or 59 or 60 or 61 or 62
64. ((“community health worker” or “community health workers” or “community health volunteer” or “community health volunteers” or “community health representative” or “community health representatives” or “community health mobilizer” or “community health mobilizers” or “community health mobiliser” or “community health mobilisers” or “community health extension worker” or “community health extension workers” or “community health aide” or “community health aides” or “community-based health worker” or “community-based health workers” or “community-based health volunteer” or “community-based health volunteers” or “community-based health representative” or “community-based health representatives” or “community-based health mobilizer” or “community-based health mobilizers” or “community-based health mobiliser” or “community-based health mobilisers” or “community-based health extension worker” or “community-based health extension workers” or “community-based health aide” or “community-based health aides” or “community based health worker” or “community based health workers” or “community based health volunteer” or “community based health volunteers” or “community based health representative” or “community based health representatives” or “community based health mobilizer” or “community based health mobilizers” or “community based health mobiliser” or “community based health mobilisers” or “community based health extension worker” or “community based health extension workers” or “community based health aide” or “community based health aides” or “barefoot doctor” or “barefoot doctors” or “village health worker” or “village health workers” or “village health volunteer” or “village health volunteers” or “village health representative” or “village health representatives” or “village health mobilizer” or “village health mobilizers” or “village health mobiliser” or “village health mobilisers” or “village health extension worker” or “village health extension workers” or “village health aide” or “village health aides” or “lay health advisor” or “lay health advisors” or “lay health educator” or “lay health educators” or “lay health home visitor” or “lay health home visitors” or “lay health leader” or “lay health leaders” or “lay health promoter” or “lay health promoters” or “lay health volunteer” or “lay health volunteers” or “lay health worker” or “lay health workers” or promotore or promotores) adj5 (view or views or reflection or reflections or reflect or reflects or value or values or valued or motivation or motivations or motivate or motivates or motivated or emotion or emotions or memory or memories or expectations or expectation or expects or expect or expected or satisfaction or satisfied or dissatisfaction or dissatisfied or disappointed or disappoint or disappointment or challenge or challenges or barriers or barrier or perspective or perspectives or experience or experiences or experienced or opinions or opinion or belief or beliefs or believes or believe or believed or attitude or attitudes or perceptions or perception or perceive or perceives or perceived or prospect or prospects or feeling or feelings or understanding)).mp. [mp=title, abstract, original title, name of substance word, subject heading word, floating sub-heading word, keyword heading word, organism supplementary concept word, protocol supplementary concept word, rare disease supplementary concept word, unique identifier, synonyms]
65. ((community or village or lay) adj3 (“change agent” or “change agents” or “health promoter” or “health promoters”)).mp. [mp=title, abstract, original title, name of substance word, subject heading word, floating sub-heading word, keyword heading word, organism supplementary concept word, protocol supplementary concept word, rare disease supplementary concept word, unique identifier, synonyms]
66. 64 or 65
67. 63 and 66

## Appendix B: Study characteristics

**Table:**

	Author(s)/year	Study title	Country	Area	Participants	Study objective	Study design	Intervention area
1	Haile et al., 2014	Assessment of non-financial incentives for volunteer community health workers – the case of Wukro district, Tigray, Ethiopia	Ethiopia	Rural	VCHVs n = 400, female	Investigate the non-financial incentives for VCHWs and factors affecting their motivation.	Cross-sectionalquantitative	Neonatal mortality
2	Maesa andKalofonosb, 2013	Becoming and remaining community health workers: Perspectives from Ethiopia and Mozambique	Ethiopia and Mozambique	Rural	Case study of 2 CHWs, a single mother in Chimoio and single man who volunteers as a CHWs	Why people become and remain CHWs, comparison between Addis Ababa and Chimoio	Qualitative:Ethnography	HIV/AIDS
3	Kelly et al., 2018	Can the financial burden of being a community health volunteer in western Kenya exacerbate poverty?	Western Kenya	Rural	2013 n = 35 CHVs 48.6% female and 51.4% male; 2015 n = 29 CHVs 58.6% female and 41.4% male	Determine how the implementation of a pooled incentive model had an impact on the lives of CHVs from two counties in western Kenya.	Qualitative	HIV/AIDS
4	Bakibinga et al., 2020	Challenges and prospects for implementation of community health volunteers’ digital health solutions in Kenya: a qualitative study	Kenya	Urban	CHVs n = 25 3 male and 22 female; Health providers and sCHMTs n = 10 4 male and 6 female	Explores the experiences of CHVs, health workers and members of Sub-County Health Management Teams following implementation of the decision support mobile health app	Qualitative	eHealth
5	Banek et al., 2014	Community case management of malaria: exploring support, capacity and motivation of community medicine distributors in Uganda	Uganda	n/a	CMDs n = 100 56% female 44% male	To understand the level of support available, and the capacity and motivation of community health workers to deliver these expanded services	Mixed	Malaria
6	Lusambili et al., 2021	Community health volunteers challenges and preferred income generating activities for sustainability: a qualitative case study of rural Kilifi, Kenya	Kenya	Rural	CHVs n = 81 64 female and 17 male	Determine challenges of volunteerism in community health and the preferred LGAs in rural Kilifi county, Kenya.	Qualitative	MCH
7	Laurenzi et al., 2020	Balancing roles and blurring boundaries: Community health workers’ experiences of navigating the crossroads between personal and professional life in rural South Africa	South Africa	Rural	CHVs (referred to as MMs) n = 10	Implications of the difficulties and burdens of being an MM on an immediate level (equipping CHWs with self‐care and boundary‐setting skills), and an intermediate level (introducing opportunities for structured debriefings and emphasizing supportive supervision).	Qualitative	MCH
8	Closser et al., 2019	Does volunteer community health work empower women? Evidence from Ethiopia’s Women’s Development Army	Ethiopia	Rural	CHVs (referred to as HEWs or WDAs) n = 69, women	Determine whether volunteer community health work empowers or burdens women	Qualitative	MCH
9	Mlotshwa et al., 2015	Exploring the perceptions and experiences of community health workers using role identity theory	South Africa	Rural	CHWs n = 18 89% female 11% male	The role identity theory framework was used to understand the work of CHWs within their communities, addressing themes, such as entry into, and nature of, caring roles, organizational support, state resourcing, and community acceptability	Qualitative	SBCC
10	Jigssa et al., 2018	Factors contributing to motivation of volunteer community health workers in Ethiopia: the case of four woredas (districts) in Oromia and Tigray regions	Ethiopia	Rural	vCHWs (1to5NL) n = 786 100% female	Examine factors contributing to the motivation of volunteer CHWs (vCHWs) in Ethiopia currently known as one-to-five network leaders (1to5NLs) and explore variations between attributes of social and work-related determinants.	Quantitative	MCH
11	Loth et al., 2020	“From good hearted community members we get volunteers”–an exploratory study of palliative care volunteers across Africa	21 countries in Africa: Botswana, Egypt, Ghana, Kenya, Malawi, Mozambique, Namibia, Rwanda, Sudan, Swaziland, Tanzania, Uganda,Zambia, Zimbabwe, Benin, Burkina Faso, Burundi, Côte d’Ivoire, DRC, Rwanda, Senegal	n/a (widevariety of locations)	n = 25 National Champions 36% female 48% male 6% no response	Determine types of volunteers, their motivations and roles in service delivery in palliative care	Mixed	Palliative Care
12	Kok et al., 2019	Getting more than “claps”: incentive preferences of voluntary community-based mobilizers in Tanzania	Tanzania	Urban	CBMs n = 17 CMB supervisors n = 11 Phase 2: CMBs n = 61	Explore the drivers of CBM motivation and inform the design of an incentive scheme.	Mixed	SBCC
13	Kok et al., 2016	Health surveillance assistants as intermediates between the community and health sector in Malawi: exploring how relationships influence performance	Malawi	Rural	FGD (HSAs, women with under 5 children, volunteers) n = 137 Semi-structured interviews n = 44 (HSAs, senior HSAss, mothers, traditional birth attendants, district level managers and health staff, health centre in charges, NGO representatives, traditional leaders, volunteers	Aimed to obtain in-depth insight into the facilitators of and barriers to interpersonal relationships between health surveillance assistants (HSAs) and actors in the community and health sector in hard-to-reach settings in two districts in Malawi, in order to inform policy and practice on optimizing HSA performance.	Qualitative	HRH
14	Zulu et al., 2014	Hope and despair: community health assistants’ experiences of working in a rural district in Zambia	Zambia	Rural	CHAs n = 12 42% women 58% male	Document motivation to become a CHA, their experiences of working in a rural district, and how these experiences affected their motivation to work	Qualitative	HRH
15	Chowdhury et al., 2021	How workers respond to social rewards: evidence from community health workers in Uganda	Uganda	Not specified	CHWs n = 4050 female	This paper investigates the effect of a non-financial incentive – a competitive annual award – on community health workers’ (CHWs) performance, an issue in the public health literature that has not been explored to its potential.	Mixed	HRH
16	Tuyisenge et al., 2020	“I cannot say no when a pregnant woman needs my support to get to the health centre”: involvement of community health workers in Rwanda’s maternal health	Rwanda	Not specified	M-CHWs n = 16 female	Explore *M*-CHWs’ perceptions and experiences on access and provision of maternal health services.	Qualitative	MCH
17	Swartz & Colvin, 2015	‘It’s in our veins’: caring natures and material motivations of community health workers in contexts of economic marginalisation	South Africa	Rural and urban	Non-specific case study of CHWs in Khayelitsha	Explore here how CHWs narrate and understand their roles and motivations as careers and members of a resource-constrained community.	Qualitative	HRH
18	Ritchie et al., 2016	Lay Health Workers experience of a tailored knowledge translation intervention to improve job skills and knowledge: a qualitative study in Zomba district Malawi	Malawi	Rural	LWHs n = 35 nurse n = 1 56% male 44% female	Explore LHWs responses to a tailored knowledge translation intervention they received, designed to address a previously identified training and knowledge gap.	Qualitative	TB
19	Mpembeni et al., 2015	Motivation and satisfaction among community health workers in Morogoro Region, Tanzania: nuanced needs and varied ambitions	Tanzania	Rural	CHWs n = 228 55% male 45% female	Study assessed motivation and satisfaction among these CHWs.	Mixed	MCH
20	Topp et al., 2015	Motivations for entering and remaining in volunteer service: findings from a mixed-method survey among HIV caregivers in Zambia	Zambia	Rural, urban and peri-urban	Caregivers n = 758 40% male 60% female	This study aimed to advance understanding of why indi-viduals in Zambia enter into and remain in volunteer service by identifying (a) motivations for volunteering(b) the extent to which these motivations are fulfilled in service and (c) factors related to discontinuation or pro-longation of service.	Mixed	HIV/AIDS
21	Nyalunga et al., 2019	Perceptions of community health workers on their training, teamwork and practice: a cross-sectional study in Tshwane district, Gauteng, South Africa	South Africa	Rural and urban	CHWs n = 431 88.2% female 11.8% male	Assess perceptions of community health workers on their training, teamwork and practice.	Cross-sectional	HIV/AIDS
22	Aseyo et al., 2018	Realities and experiences of community health volunteers as agents for behaviour change: evidence from an informal urban settlement in Kisumu, Kenya	Kenya	Peri-urban	CHVs n = 16 69% female 31% male	Examine the behaviour change-related activities of community health volunteers (CHVs)—community health workers affiliated with the Kenyan Ministry of Health – in a peri-urban settlement in Kenya, in order to assess their capabilities, opportunities to work effectively, and sources of motivation	Mixed	HRH
23	Ormel et al., 2019	Salaried and voluntary community health workers: exploring how incentives and expectation gaps influence motivation	Ethiopia, Kenya, Malawi and Mozambique	Rural	n not specified, 250 interviews and 65 FGDs conducted	Critically analyse the use of incentives and their link with improving CHW motivation.	Qualitative	HRH
24	Peltzer et al., 2014	Secondary trauma and job burnout and associated factors among HIV lay counsellors in Nkangala district, South Africa	South Africa	Not specified	HIV lay counsellors n = 117 91.6% female 8.4% male	Evaluate secondary trauma and job burnout and associated factors in a sample of 71 HIV lay counsellors in South Africa.	Mixed	HIV/AIDS
25	Greenspan et al., 2013	Sources of community health worker motivation: a qualitative study in Morogoro Region, Tanzania	Tanzania	Rural	CHWs n = 20 50% male 50% female	Explore sources of CHW motivation to inform programs in Tanzania and similar contexts.	Qualitative	HRH
26	Jack et al., 2011	The personal value of being a palliative care Community Volunteer Worker in Uganda: A qualitative study	Uganda	Rural	Hospice Clinical Teams n = 11, CVWs n = 32 41% male 53% female 6% not reported	The aim of this study was to evaluate the motivation for becoming a volunteer and the personal impact of being a palliative care Community Volunteer Worker in Uganda.	Qualitative	Palliative Care
27	Sam-Agudu et al., 2018	“They do not see us as one of them”: a qualitative exploration of mentor mothers ‘working relationships with healthcare workers in rural North-Central Nigeria	Nigeria	Rural	MMs n = 36 female	Explore the experiences and opinions of MMs with respect to their work conditions and relationships with health care workers.	Qualitative	HIV/AIDS
28	Maesa 2014	“Volunteers Are Not Paid Because They Are Priceless”: Community Health Worker Capacities and Values in an AIDS Treatment Intervention in Urban Ethiopia	Ethiopia	Urban	CHW n = 110 90% female 10% male	Analyze community health workers’ (CHW) capacities for empathic service within an AIDS treatment program in Addis Ababa.	Qualitative	HIV/AIDS
29	O’Donovan et al. (2020)	‘We are the people whose opinions don’t matter’. A photovoice study exploring challenges faced by community health workers in Uganda	Uganda	Rural	CHWs n = 8 50% male 50% female	Understand the experiences of community health workers (CHWs) through the use of participatory visual methods (PVMs)	Qualitative	HRH
30	Takasugi and Lee (2012)	Why do community health workers volunteer? A qualitative study in Kenya	Kenya	Rural	CHWs n = 23 65% female 35% male	Determine factors that motivate volunteers	Qualitative	HRH
31	Kweku et al. 2020	Volunteer responsibilities, motivations and challenges in implementation of the community-based health planning and services (CHPS) initiative in Ghana: qualitative evidence from two systems learning districts of the CHPS+ project.	Ghana	Rural	CHVs +37		Qualitative	HRH

## Appendix C: Mapping studies to themes

**Table:**

Coding	Sub-themes	Themes
Taking on an extra farm or domestic work	Family obligations	Challenge of Balancing Responsibilities with Family Obligations
Inability to provide	Reason for volunteering	Limited Resources
Pressures on income-earning activities	Motivation	Stigma and Harassment
Commitment to helping their families and community	Moral/social distress	Potential gendered benefits and gendered risks
Additional financial burdens	No job aids/resources	Health system challenges
Stock out of medicines and inconsistent supply of resources	Identification and trust	
The desire for career advancement	Relationship with community	
Differences in male and female motivation to volunteer	Feeling marginalized	
Male career advancement aspirations with some harbouring political ambitions	Gendered differences	
Frustration and disappointment with the inability to achieve their desired financial objectives	Safety	
Male CHVs regarded volunteering as a pathway to higher positions	Supervision and management	
Feeling unsafe while volunteering	Skills and knowledge (capacity)	
Absence or lack of standardized remuneration	Motivation	
Partial financial incentives	Government remuneration/financial compensation	
Increased and unclear scope of work; unclear division of labour		
Inequity of their non-remuneration		
